# Draft Genome Sequence of Bioactive Strain Streptomyces sp. SMS_SU21, Isolated from Soil Sediment of the Sundarbans Mangrove Ecosystem

**DOI:** 10.1128/genomeA.00614-18

**Published:** 2018-07-05

**Authors:** Sohan Sengupta, Arnab Pramanik, Pijush Basak, Maitree Bhattacharyya

**Affiliations:** a Jagadis Bose National Science Talent Search, Kolkata, West Bengal, India; b Department of Biochemistry, University of Calcutta, Kolkata, West Bengal, India

## Abstract

Streptomyces sp. SMS_SU21 possesses strong antimicrobial activity and antioxidant potential. This strain was isolated from the Sundarbans mangrove ecosystem, and its draft genome comprises 7,449,420 bp with 6,680 open reading frames. Genome analysis of strain SMS_SU21 provides insight into its secondary metabolite arsenal and reveals the gene clusters putatively responsible for its bioactive potential.

## Abstract (precis)

Streptomyces sp. SMS_SU21 possesses strong antimicrobial activity and antioxidant potential.

## GENOME ANNOUNCEMENT

Mangrove streptomycetes are rich sources of natural products with significant biological activities and novel structures (1). Genome mining of mangrove streptomycetes accelerates the rapid discovery of useful products originating from them. In this study, Streptomyces sp. SMS_SU21 was isolated from the soil sediment of the Sundarbans mangrove ecosystem in India. This strain possesses potent antimicrobial activity against a broad spectrum of microorganisms, including multidrug-resistant strains and various phytopathogens (2). Interspecies competition within the residing microbial population is an obvious phenomenon in the Sundarbans, due to the rich index of species diversity and limited consumable nutrient sources (3) found there. Thus, in-depth information regarding the genomic edifice of this strain is required to understand its survival strategies in a competitive environment like the Sundarbans mangrove ecosystem.

Genomic DNA was extracted with a HiPurA streptomycetes DNA isolation and purification kit (Himedia, India). Shotgun sequencing was performed with a high-throughput HiSeq platform (Illumina) at AgriGenome Labs Private Limited in Kerala, India. Prior to whole-genome analysis, Cutadapt version 1.8 (4) was used to remove adapter sequences, and all low-quality data (Q < 30) were filtered out using Sickle version 1.33 (5). The cleaned reads were subjected to analysis with Kmergenie (6) to predict the optimal *k*-value and assembly size, which were found to be 31 and 7,449,420 bp, respectively. *De novo* assembly was performed using SPAdes version 3.9.0 (7), Velvet (8), and QUAST (9). The genome sequence of Streptomyces sp. SMS_SU21 was annotated using the Rapid Annotations using Subsystems Technology (RAST) server (10) and the NCBI Prokaryotic Genome Annotation Pipeline (PGAP) version 4.3 (11). The draft genome sequence of Streptomyces sp. SMS_SU21 constituted a total of 93 contigs (>1,000 bp), with a total size of 7,449,420 bp and a G+C content of 72.3%. The RAST server predicted 6,680 coding sequences, of which 2,208 (34%) were annotated as SEED subsystem features and 4,472 (66%) were annotated as outside the SEED subsystem; 3 rRNAs and 67 tRNAs were also predicted. The closest related type strains based on the 16S rRNA gene sequence are S. griseorubens NBRC 12780 (GenBank accession number AB184139), S. althioticus NRRL B-3981 (GenBank accession number AY999791), and S. griseoincarnatus LMG 19316 (GenBank accession number AJ781321), all with 99% sequence identity.

Secondary metabolite biosynthetic gene clusters (BGCs) were predicted using antiSMASH version 4.0 (12), which identified 24 putative BGCs in the genome. This includes nonribosomal peptide synthetase (NRPS) gene clusters, polyketide synthase (PKS), novel hybrid PKS-NRPS gene clusters, and other BGCs for producing siderophores, lantipeptides, lassopeptide, and bacteriocin. Numerous genes responsible for resistance to toxic compounds, including arsenic, mercury, cobalt, tellurium, and cadmium, were additionally detected. Hence, Streptomyces sp. SMS_SU21 may have great potential to produce exclusive bioactive natural compounds for clinical, industrial, and environmental applications.

### Accession number(s).

This whole-genome shotgun project has been deposited at DDBJ/ENA/GenBank under the accession number PNRA00000000. The version described in this paper is the second version, PNRA02000000.

## References

[1] Xiao J, Wang Y, Luo Y, Xie S-J, Ruan J-S, Xu J 2009 *Streptomyces avicenniae* sp. nov., a novel actinomycete isolated from the rhizosphere of the mangrove plant *Avicennia mariana*. Int J Syst Evol Microbiol 59:2624–2628. doi:10.1099/ijs.0.009357-0. 19625443

[2] Sengupta S, Pramanik A, Ghosh A, Bhattacharyya M 2015 Antimicrobial activities of actinomycetes isolated from unexplored regions of Sundarbans mangrove ecosystem. BMC Microbiol 15:170. doi:10.1186/s12866-015-0495-4. 26293487 PMC4546244

[3] Basak P, Majumder NS, Nag S, Bhattacharyya A, Roy D, Chakraborty A, SenGupta S, Roy A, Mukherjee A, Pattanayak A, Ghosh A, Chattopadhyay D, Bhattacharyya M 2015 Spatiotemporal analysis of bacterial diversity in sediments of Sundarbans using parallel 16S rRNA gene tag sequencing. Microb Ecol 69:500–511. doi:10.1007/s00248-014-0498-y. 25256302

[4] Martin M 2011 Cutadapt removes adapter sequences from high throughput sequencing reads. EMBnet J 17:10–12. doi:10.14806/ej.17.1.200.

[5] Joshi NA, Fass JN 2011 Sickle: a sliding-window, adaptive, quality-based trimming tool for FastQ files (version 1.33). https://github.com/najoshi/sickle.

[6] Chikhi R, Medvedev P 2014 Informed and automated *k*-mer size selection for genome assembly. Bioinformatics 30:31–37. doi:10.1093/bioinformatics/btt310. 23732276

[7] Bankevich A, Nurk S, Antipov D, Gurevich AA, Dvorkin M, Kulikov AS, Lesin VM, Nikolenko SI, Pham S, Prjibelski AD, Pyshkin AV, Sirotkin AV, Vyahhi N, Tesler G, Alekseyev MA, Pevzner PA 2012 SPAdes: a new genome assembly algorithm and its applications to single-cell sequencing. J Comput Biol 19:455–477. doi:10.1089/cmb.2012.0021. 22506599 PMC3342519

[8] Zerbino DR 2010 Using the velvet *de novo* assembler for short-read sequencing technologies. Curr Protoc Bioinformatics 11:1–13. doi:10.1002/0471250953.bi1105s31. PMC2952100 20836074

[9] Gurevich A, Saveliev V, Vyahhi N, Tesler G 2013 QUAST: quality assessment tool for genome assemblies. Bioinformatics 29:1072–1075. doi:10.1093/bioinformatics/btt086. 23422339 PMC3624806

[10] Aziz RK, Bartels D, Best A, DeJongh M, Disz T, Edwards RA, Formsma K, Gerdes S, Glass EM, Kubal M, Meyer F, Olsen GJ, Olson R, Osterman AL, Overbeek RA, McNeil LK, Paarmann D, Paczian T, Parrello B, Pusch GD, Reich C, Stevens R, Vassieva O, Vonstein V, Wilke A, Zagnitko O 2008 The RAST server: Rapid Annotations using Subsystems Technology. BMC Genomics 9:75. doi:10.1186/1471-2164-9-75. 18261238 PMC2265698

[11] Tatusova T, DiCuccio M, Badretdin A, Chetvernin V, Nawrocki EP, Zaslavsky L, Lomsadze A, Pruitt KD, Borodovsky M, Ostell J 2016 NCBI Prokaryotic Genome Annotation Pipeline. Nucleic Acids Res 44:6614–6624. doi:10.1093/nar/gkw569. 27342282 PMC5001611

[12] Blin K, Wolf T, Chevrette MG, Lu X, Schwalen CJ, Kautsar SA, Suarez Duran HG, de los Santos ELC, Kim HU, Nave M, Dickschat JS, Mitchell DA, Shelest E, Breitling R, Takano E, Lee SY, Weber T, Medema MH 2017 antiSMASH 4.0—improvements in chemistry prediction and gene cluster boundary identiﬁcation. Nucleic Acids Res 45:W36–W41. doi:10.1093/nar/gkx319. 28460038 PMC5570095

